# Problem-based learning promotes causal physiological reasoning in first-year medical students before formal cardiovascular instruction

**DOI:** 10.3389/fphys.2026.1897473

**Published:** 2026-07-17

**Authors:** Carmen Morales-Luque, Marta González-García, Laura Carrillo-Franco, Marc Stefan Dawid-Milner, Belén Gago, Manuel Víctor López-González

**Affiliations:** 1 Department of Human Physiology, Faculty of Medicine, University of Malaga, Malaga, Spain; 2 Unit of Neurophysiology of the Autonomic Nervous System (CIMES), University of Malaga, Malaga, Spain; 3 IBIMA Plataforma BIONAND, Malaga, Spain; 4 Department of Nursing, Faculty of Health Sciences, University of Malaga, Malaga, Spain

**Keywords:** causal reasoning, clinical reasoning, physiology education, preclinical medical education, problem-based learning, undergraduate medical education

## Abstract

Teaching Human Physiology in undergraduate medical education requires more than the acquisition of conceptual knowledge; it demands that students develop causal explanatory frameworks linking physiological mechanisms to their clinical manifestations. Problem-Based Learning (PBL) has been proposed as a strategy to promote this integrative form of reasoning, yet evidence in preclinical physiology, particularly among first-year medical students without prior formal clinical exposure, remains limited. This quasi-experimental pre–post study evaluated the impact of a two-session PBL intervention on physiological reasoning in 135 first-year medical students at the University of Málaga. The intervention comprised six contact hours delivered across five small-group classes (25–30 students per class) organized into teams of five students and was triggered by two real cardiovascular events involving elite athletes. Written responses to two open-ended questions administered before (PRE) and after (POST) the intervention were coded using an ordinal framework (N0–N4) that independently assessed mechanistic conceptual complexity (P1: physiological model of the heart as an effective pump) and physiological cause–effect integration (P2: physiological rationale underlying healthcare intervention following loss of effective blood flow). Both dimensions improved significantly after the intervention. The effect on physiological cause–effect integration was large (P2: Z = −7.723, p < 0.001, r = 0.68), with the proportion of students reaching advanced integrative reasoning levels (N3–N4) increasing from 3.8% to 53.8%. The effect on mechanistic conceptual complexity was moderate (P1: Z = −4.683, p < 0.001, r = 0.41). Improvements in the two dimensions were independent of one another (rs = 0.128, p > 0.05), indicating that they represent distinct cognitive constructs. These findings suggest that brief, carefully designed PBL sequences anchored in authentic clinical problems can produce substantial gains in causal physiological reasoning among first-year medical students, even before the formal introduction of cardiovascular physiology in lecture-based teaching.

## Highlights

- Brief PBL exposure produced substantial gains in causal physiological reasoning before formal cardiovascular physiology instruction.
- Physiological cause–effect integration showed a large educational effect (P2: r = 0.68), exceeding gains in mechanistic conceptual complexity (P1: r = 0.41).
- Mechanistic and causal dimensions of physiological reasoning improved independently, indicating distinct developmental trajectories.
- The N0–N4 coding framework enables systematic assessment of conceptual progression in physiology education.

## 1 Introduction

Teaching Physiology in undergraduate medical education presents a unique pedagogical challenge. Beyond acquiring a repertoire of concepts describing how the human body functions, students must learn to organize this knowledge into causal explanatory frameworks that connect physiological mechanisms with their clinical manifestations. This integrative dimension—understanding why a phenomenon occurs rather than merely what occurs—constitutes the foundation of clinical reasoning and represents one of the most difficult educational goals to achieve during the preclinical years (Langer et al., 2021; Lira et al., 2024). Qualitative studies conducted in preclinical settings indicate that clinical reasoning instruction is often delivered implicitly, without a shared vocabulary or explicit modelling of the underlying causal processes, leading to instructional inconsistency and increased cognitive load for learners (Maciuba et al., 2023).

Traditional lecture-based approaches, centered on the systematic transmission of content, have shown limitations in developing this capacity in a robust and durable manner (Csaba et al., 2025). Physiological knowledge acquired through conventional teaching has been reported to decline by approximately 25% within the first 16 weeks after assessment when active consolidation strategies are not implemented (Csaba et al., 2025). In first-year physiology education specifically, comparative studies have documented substantially higher knowledge-retention rates with inquiry-based learning (64–100%) than with team-based learning (14–38%), suggesting that the type of active-learning strategy influences the long-term retention of physiological knowledge (Gaddas et al., 2026).

Problem-Based Learning (PBL) has emerged as one of the most widely adopted pedagogical approaches in medical education. In PBL, students are confronted with a clinical case or authentic problem scenario before receiving direct instruction, stimulating autonomous inquiry, hypothesis generation, and collaborative knowledge construction within small-group settings (Li et al., 2022; Abu Elgasim et al., 2025). Recent reviews indicate that PBL is associated with improvements in perceived critical-thinking and communication skills among undergraduate medical students (Abugri et al., 2026), higher student satisfaction and self-directed learning behaviors in postgraduate medical education without compromising knowledge acquisition (Mensour et al., 2025), and significant gains in critical thinking and clinical reasoning among nursing students (Sharma et al., 2023; Oliveira Silva et al., 2025).

Evidence suggests that educational strategies promoting early exposure to authentic clinical problems not only enhance student motivation and engagement but also facilitate the organization of basic biomedical knowledge into cognitive structures oriented toward causal explanation (Langer et al., 2021; Vieira, 2025). Recent studies in health sciences education further indicate that active-learning approaches increase student engagement with content that might otherwise be perceived as abstract, thereby fostering cognitive integration between foundational scientific knowledge and clinical context (Jooste and Shaikh-Kader, 2026; Karami and Hussein, 2026). This effect appears particularly pronounced when authentic clinical scenarios are used to trigger students’ independent inquiry processes.

Despite these advantages, evidence regarding the impact of PBL in General Physiology and Human Physiology courses during the first year of medical training remains limited. Much of the available literature in basic medical education relies on conventional outcome measures, such as examination grades or multiple-choice tests, or on students’ subjective perceptions, without directly assessing the complexity and causal coherence of physiological reasoning (Sharma et al., 2023; Mensour et al., 2025). When learning outcomes are evaluated using instruments that are insensitive to causal integration, PBL tends to produce gains in declarative knowledge comparable to those achieved through traditional instruction (Mensour et al., 2025). Likewise, measures of self-reported confidence in clinical reasoning provide only a partial representation of learning outcomes (Kirchberg et al., 2026). These methodological limitations hinder the ability to determine the extent to which PBL promotes the conceptual integration that characterizes deep learning in physiology.

Assessing the organization of physiological knowledge requires instruments capable of capturing not only the quantity of concepts available to students but also the causal architecture linking those concepts. One example is the use of concept maps generated during information-search and synthesis processes, which allow visualization and evaluation of students’ knowledge structure and integration while facilitating the identification of conceptual connections, gaps, and misconceptions (Fonseca et al., 2024; Shanmugarajah et al., 2024; Aljamal et al., 2026). Supporting this perspective, a recent systematic review reported moderate-to-large effect sizes (d = 0.7–0.8) in studies employing concept mapping among preclinical medical students (Aljamal et al., 2026), consistent with the principle of knowledge externalization that underlies other visual tools specifically developed for learning cardiovascular physiology (Lujan and DiCarlo, 2025). Nevertheless, most systems used to analyze these student-generated products lack sufficient depth to discriminate among different levels of causal integration, thereby limiting the sensitivity of pre–post comparisons and the identification of individual learning trajectories.

Unlike studies that evaluate PBL primarily through aggregated outcome measures, the present work adopts a conceptual-change perspective focused on the dynamics of learning progression. The central question is not merely whether students improve, but how they move across hierarchically defined levels of physiological reasoning, which transitions occur most frequently, and which individual learning patterns underlie the overall distribution of outcomes. Accordingly, this study evaluated the impact of a PBL intervention on physiological reasoning among 135 first-year medical students enrolled in the cardiovascular physiology module of the Human Physiology course at the University of Málaga. The intervention was based on a process of student-directed inquiry facilitated by instructors in small-group classes (25–30 students) and structured around an authentic clinical trigger. Its impact was assessed through the analysis of pre–post responses to open-ended questions using an ordinal coding framework that independently evaluated mechanistic conceptual complexity in cardiovascular physiology and physiological cause–effect integration within healthcare intervention reasoning. The specific objectives were to: (1) determine whether the intervention produced significant improvements in both dimensions; (2) estimate the magnitude of these effects; (3) characterize individual patterns of conceptual change through transition matrices; and (4) describe students’ perceptions of the intervention.

## 2 Materials and methods

### 2.1 Study design and educational context

A descriptive-analytical quasi-experimental study with a pre–post design was conducted to evaluate conceptual development among first-year medical students at the University of Málaga following a contextualized Problem-Based Learning (PBL) intervention integrated into the cardiovascular physiology module of the Human Physiology I course.

All participants (n = 135) engaged in the activity for the first time and had no prior experience with structured PBL methodologies. At the time of the PRE assessment, students had not yet received formal lecture-based instruction on cardiovascular physiology. Consequently, their understanding was limited to intuitive notions derived from previous pre-university education. Participants had not received formal training in emergency healthcare interventions, the physiological basis of cardiopulmonary resuscitation (CPR), or cardiovascular pathophysiology before the intervention.

The educational activity was delivered across five small-group classes comprising 25–30 students each. Within each class, students worked in cooperative teams of five members. No independent control group was included. During the intervention period, students simultaneously attended conventional lecture-based teaching covering the cardiovascular physiology module. Therefore, the primary aim of the study was to analyze changes in the conceptual organization and causal complexity of students’ physiological explanations, as well as patterns of transition between levels of physiological reasoning. Consequently, observed changes cannot be attributed exclusively to the PBL intervention but rather to the overall contextualized educational sequence implemented throughout the module.

### 2.2 Ethical considerations

The study was approved by the Ethics Committee of the University of Málaga (CEUMA; reference no. 187-2024-H). Before completing the PRE assessment, all students received information regarding the educational and research use of data generated during the activity. Participation was conditional upon explicit electronic informed consent provided through a binary (“yes/no”) response integrated into the activity platform. Only students who provided consent were included in the analyses. All responses were anonymized prior to coding and statistical evaluation.

### 2.3 Problem-based learning sequence

**Figure 1:**
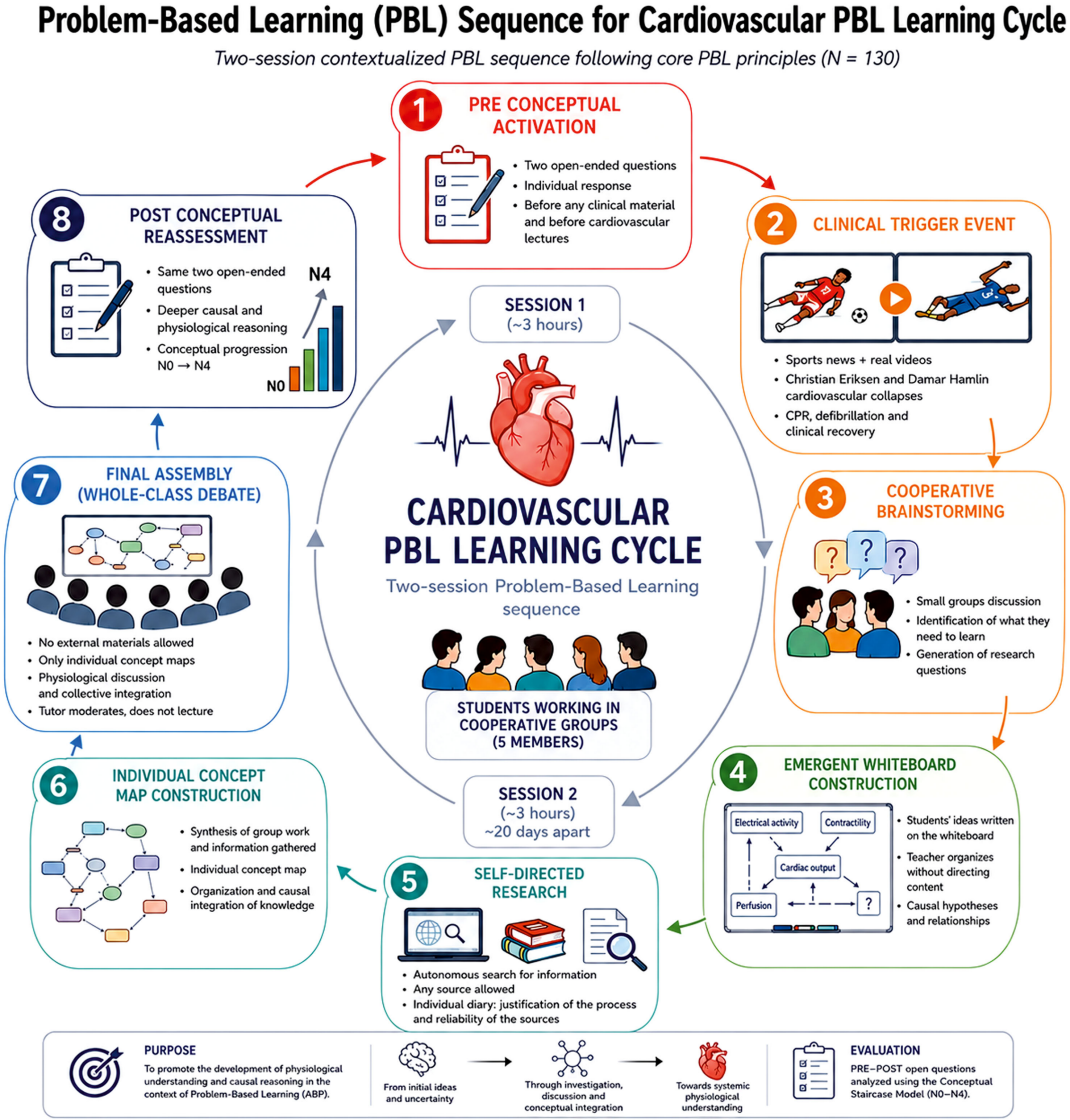
Problem-based learning sequence and learning cycle implemented in the cardiovascular PBL intervention. Schematic representation of the two-session Problem-Based Learning (PBL) intervention implemented in first-year medical students. The sequence began with an individual pre-intervention assessment of physiological reasoning, followed by presentation of a real cardiovascular emergency scenario involving elite athletes, collaborative brainstorming, teacher-facilitated whiteboard construction, autonomous information seeking, individual concept-map development, and whole-class discussion. The process concluded with a post-intervention reassessment using the same open-ended questions. The diagram illustrates the progression from initial uncertainty to integrated physiological understanding through collaborative inquiry and self-directed learning.

The intervention consisted of a contextualized PBL sequence delivered in two face-to-face sessions of three hours each, separated by a three-week interval. The instructional design (**Figure 1**) followed the core principles of PBL: (1) presentation of an authentic problem situation before direct instruction, (2) student-generated learning questions, (3) independent information searching and critical appraisal of sources, (4) individual synthesis through concept mapping, and (5) a final plenary discussion (Li et al., 2022).

During the first session, following a brief introduction to the general principles of PBL, students individually answered two open-ended PRE questions designed to explore their initial understanding of:

1. The physiological mechanisms that enable the heart to function as an effective pump.
2. The physiological rationale underlying healthcare intervention following the loss of effective blood flow.

The PRE responses were completed before exposure to any clinical material and prior to formal lecture-based teaching of the cardiovascular physiology module. Students were then presented with a trigger scenario consisting of a sports news article developed by the instructors, accompanied by authentic video recordings of the cardiovascular collapses experienced by professional athletes Christian Eriksen and Damar Hamlin during competition. Both cases contained explicit contextual information related to cardiac arrest, cardiopulmonary resuscitation, defibrillation, and subsequent clinical recovery.

Working in cooperative groups, students identified the physiological knowledge required to explain the observed events. Each small-group class generated its own research questions, which were organized by the instructors on a whiteboard without directing the conceptual content proposed by students (example provided in **Supplementary Material 1**).

**Figure 2:**
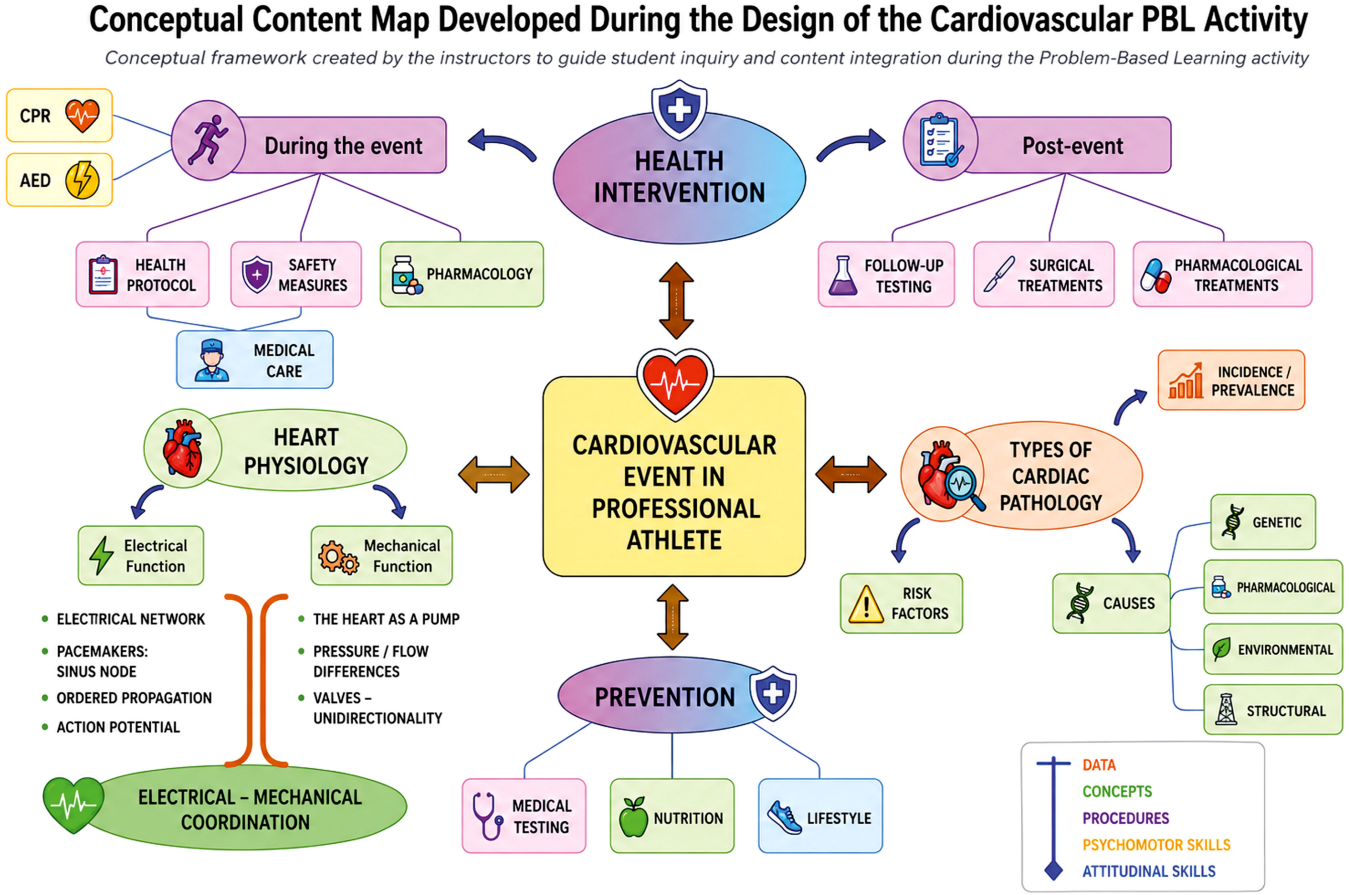
Conceptual content map developed by the teaching staff during the design of the cardiovascular PBL activity. Conceptual framework created by the instructors to define the scope and structure of the learning process before implementation of the intervention. The map integrates the main thematic domains addressed throughout the activity, including cardiovascular physiology, cardiac pathology, prevention strategies, emergency management, medical intervention, and post-event care in professional athletes. Different colors indicate the educational dimensions incorporated into the intervention, including factual knowledge, conceptual understanding, procedures, psychomotor skills, and attitudinal competencies.

During the self-directed learning phase, students were allowed to consult any information source, provided that they justified their selection and evaluated its reliability in an individual learning diary. Each student subsequently produced an individual concept map integrating information gathered during both group discussion and independent inquiry. Both the learning diary and the concept map contributed to course assessment. The content map generated by the instructors during the design of the activity (**Figure 2**) was subsequently compared with the concept maps produced by students (example provided in **Supplementary Material 2**).

The second session consisted of a plenary discussion focused on the physiological interpretation and integration of the concepts investigated. Students participated without access to external resources and relied exclusively on their own concept maps. Immediately after the discussion, participants completed the same two open-ended questions in POST format and provided a written personal reflection on the activity.

### 2.4 Assessment instruments

#### 2.4.1 Pre–post open-ended questions

The following questions were administered before and after the intervention:

- Question 1 (P1): “Using your own words, explain what enables the heart to function as an effective pump capable of maintaining continuous blood circulation.”
- Question 2 (P2): “If the heart fails to maintain effective blood flow, explain how healthcare providers should intervene and what physiological objective such intervention seeks to achieve.”

P1 exclusively assessed students’ conceptual physiological model of the heart as a functional pump, whereas P2 explored pathophysiological reasoning and understanding of the physiological rationale underlying healthcare intervention in situations of cardiac arrest or critical perfusion failure.

The distinction between P1 and P2 reflects the difference between proximate-How reasoning (mechanistic description of the cardiac pump model) and proximate-Why reasoning (physiological cause–effect integration underlying healthcare intervention), dimensions that physiology education research has shown to be differentially activated by specific instructional prompts and scaffolding strategies (Lira et al., 2024; Pelkowski et al., 2025; Wafi, 2025).

### 2.5 Conceptual coding framework

PRE and POST responses were analyzed using an ordinal coding framework specifically developed for this study to evaluate conceptual complexity and causal integration within physiological explanations. Coding was performed by three evaluators. Two independent raters (A and B) coded all responses while blinded to one another’s assessments, and a third evaluator (C) acted as an adjudicator, resolving discrepancies and assigning the final score.

**Figure 3:**
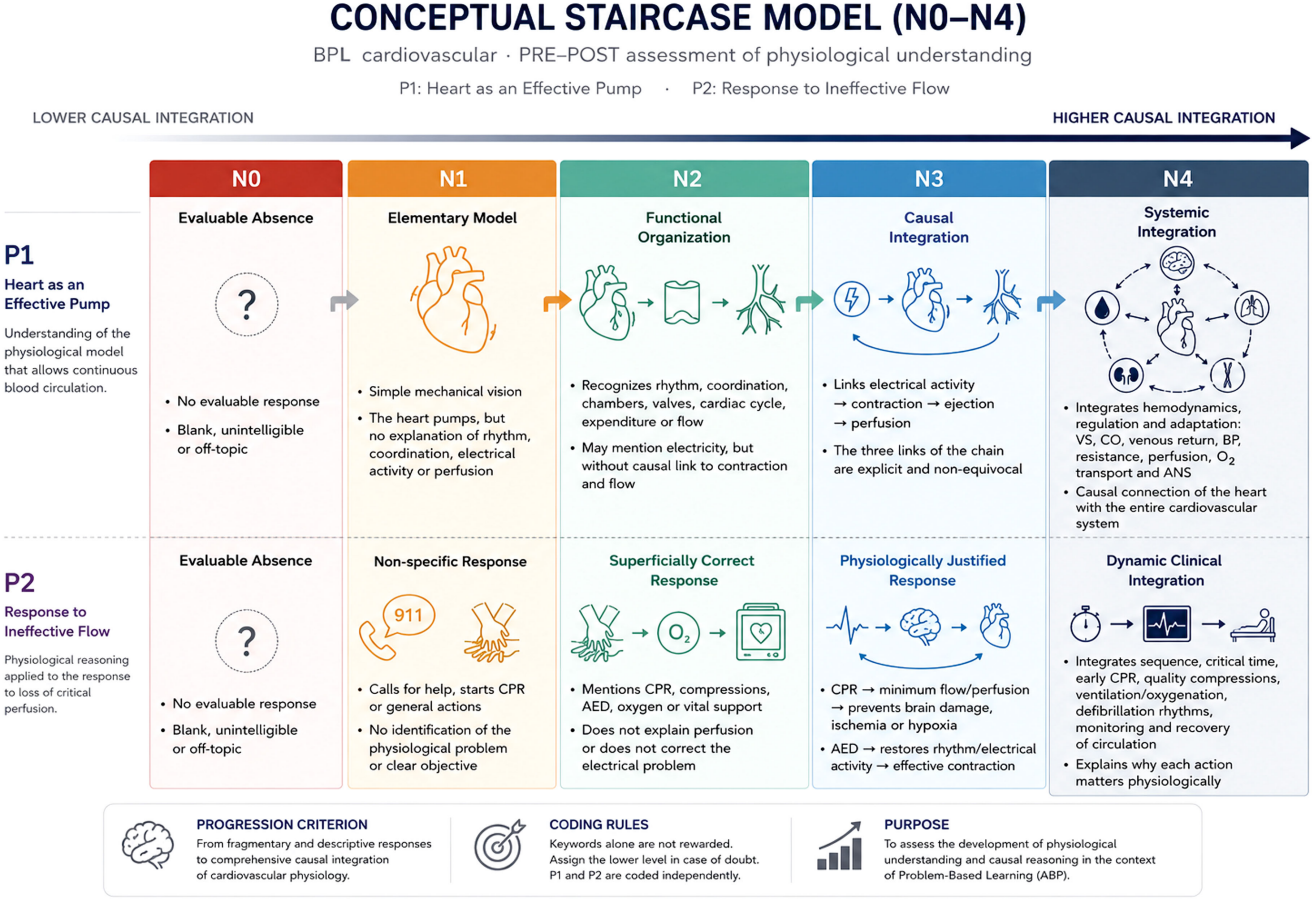
Conceptual staircase model used to classify students’ physiological reasoning. Hierarchical framework defining five levels of conceptual understanding (N0–N4) applied independently to both assessment questions. For P1, levels represent increasing sophistication in explaining how the heart functions as an effective pump, progressing from non-evaluable responses (N0) to integrated systemic cardiovascular reasoning (N4). For P2, levels represent increasing physiological understanding of the purpose of emergency interventions in ineffective circulation, ranging from non-specific actions (N1) to advanced causal and clinical integration (N4). The model served as the coding framework for qualitative analysis of all PRE and POST responses.

Responses were classified into five hierarchical levels (N0–N4) according to the degree of explicit physiological complexity and causal integration demonstrated (**Figure 3**):

- NC: no evaluable response.
- N0: response present but not evaluable.
- N1: simple mechanistic view.
- N2: functional organization without explicit causal integration.
- N3: explicit electro-mechanical causal integration.
- N4: dynamic systemic physiological integration.

The use of ordinal scales to assess reasoning in written responses from medical students is well established in educational assessment research, particularly in studies evaluating problem representation and diagnostic reasoning coherence (Valentine et al., 2022; Fonseca et al., 2024). As in these frameworks, coding levels increased only when causal relationships were explicitly integrated into students’ explanations.

The hierarchical structure of the N0–N4 framework is theoretically grounded in the distinction between proximate-mechanistic and proximate-functionalist forms of physiological reasoning articulated by Lira et al. (2024), who demonstrated that these two varieties of physiological knowledge respond differently to assessment scaffolds and instructional prompts. The present framework operationalizes this distinction as an ordinal progression: levels N1–N2 capture increasing mechanistic conceptual complexity (proximate-How reasoning), while levels N3–N4 require the explicit integration of causal links connecting physiological mechanisms to their functional consequences (proximate-Why reasoning).

P1 and P2 were coded independently because they assessed different physiological constructs. The framework was designed to prevent isolated biomedical terminology from artificially inflating conceptual scores. Technical terms were considered relevant only when embedded within explicit and physiologically coherent causal explanations. In ambiguous cases, the lower coding level was assigned unless causal integration was clearly articulated.

### 2.6 Textual quality

In addition to conceptual level, overall textual quality was assessed using a four-level ordinal scale:

1. Poor.
2. Basic but understandable.
3. Clear and well-structured.
4. Highly precise.

Textual quality was treated as an independent dimension, allowing differentiation between physiological understanding and written expression. This measure was included to distinguish conceptual progression from purely rhetorical or communicative improvement.

### 2.7 Student reflections

Written reflections collected after the intervention were analyzed using predefined thematic categories related to:

- Overall attitude toward the activity.
- Perceived learning usefulness.
- Group work.
- Usefulness of concept mapping.
- Autonomy in information searching.
- Perceived workload or difficulty.
- Organization and implementation of the activity.

Each category was coded as positive (+1), neutral (0), or negative (−1) only when such evaluation was explicitly stated in the text. Because students were aware that instructors would have access to these reflections, positive comments were interpreted cautiously due to the potential influence of social desirability bias.

### 2.8 Statistical analysis

Given the ordinal nature of the conceptual coding framework (N0–N4), analyses focused primarily on descriptive and transition-based approaches rather than assuming linear equivalence between adjacent categories. The main outcomes examined were:

- Distribution of conceptual levels in PRE and POST assessments.
- Patterns of transition between conceptual levels.
- Individual ordinal changes (Δ PRE–POST).
- Proportion of students reaching explicit causal-integration levels (N3–N4).
- Changes in textual quality.

PRE and POST distributions for P1 and P2 were summarized using absolute frequencies and percentages. Individual transition matrices were generated to characterize patterns of progression, stability, and regression across conceptual levels. Responses classified as NC in either PRE or POST assessments were excluded from analyses of individual change (Δ), as NC represented the absence of an evaluable response rather than a conceptual category.

Overall ordinal changes between PRE and POST assessments were evaluated using the Wilcoxon signed-rank test for paired samples, an appropriate non-parametric procedure for ordinal data and non-normal distributions. These analyses were interpreted as indicators of global directional change rather than evidence of equal intervals between adjacent categories. Effect size was calculated using the coefficient r derived from the Wilcoxon Z statistic and interpreted conventionally as small (r ≈ 0.1), moderate (r ≈ 0.3), or large (r ≥ 0.5). Statistical significance was established at p < 0.05. All analyses were performed using IBM SPSS Statistics version 28.

## 3 Results

### 3.1 Sample included in the analysis

A total of 135 first-year medical students participated in the educational intervention. Analyses of conceptual progression included only students who provided valid PRE and POST responses for both questions (P1 and P2). After excluding cases classified as NC in any of the variables analyzed, the final sample comprised 130 students (96.3%). All 135 participants were included in the analyses of textual quality and student perceptions of the methodology.

### 3.2 Inter-rater reliability

Inter-rater reliability between evaluators A and B was assessed using unweighted Cohen’s kappa, as the primary objective was to identify categorical disagreement rather than quantify its magnitude, treating all discrepancies equally regardless of ordinal distance. Kappa values were as follows: PRE_P1, κ = 0.44; POST_P1, κ = 0.39; PRE_P2, κ = 0.52; and POST_P2, κ = 0.43. These values (κ = 0.39–0.52) are comparable to those reported for ordinal instruments assessing clinical reasoning in similar educational contexts (κ = 0.41) (Valentine et al., 2022) and justified the involvement of a third adjudicating evaluator to resolve disagreements.

### 3.3 Distribution of conceptual levels in P1 and P2

#### 3.3.1 P1 – physiological model of the heart as an effective pump

**Figure 4:**
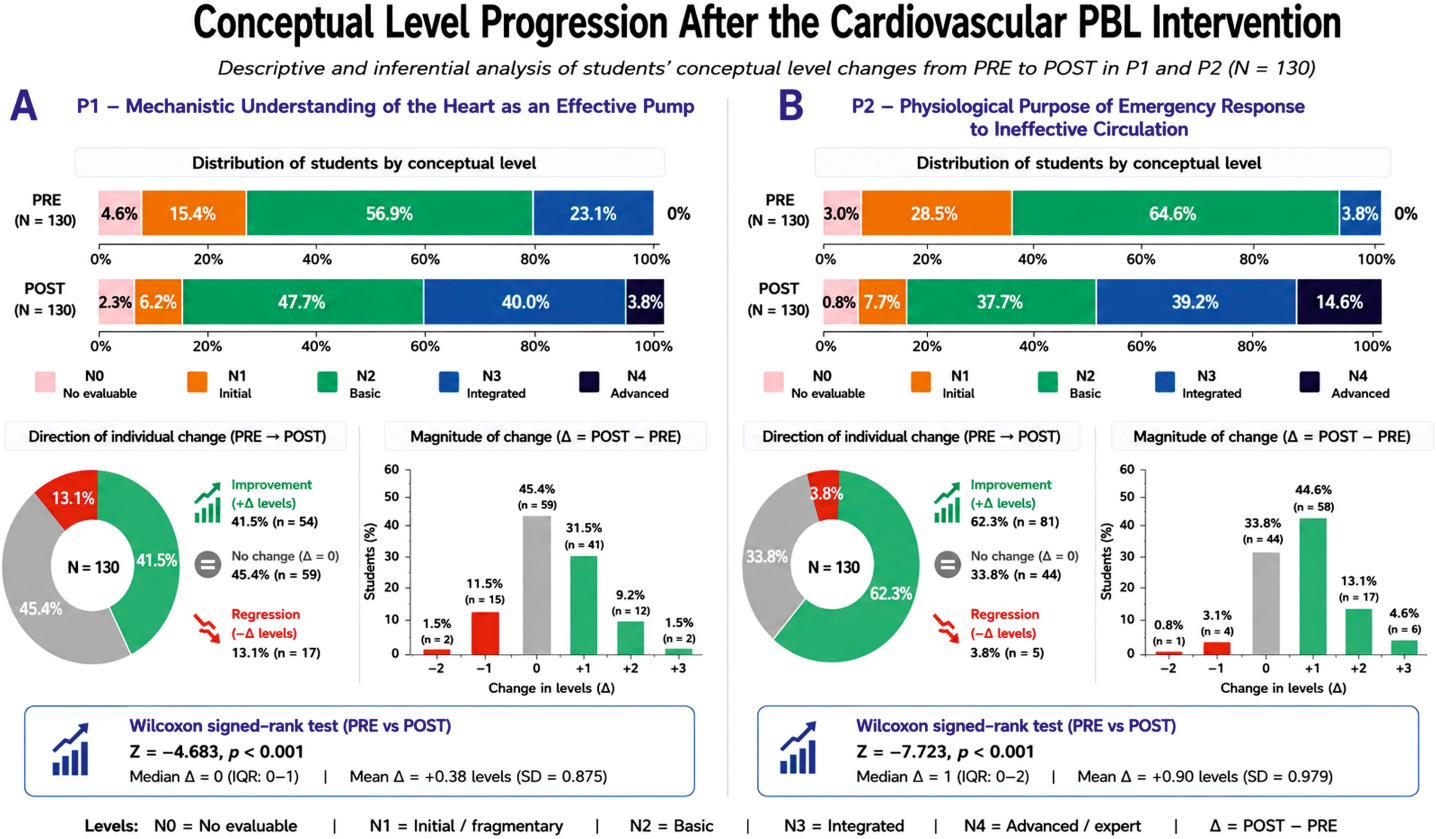
Conceptual level progression after the cardiovascular PBL intervention. Descriptive and inferential analysis of changes in students’ conceptual levels between PRE and POST assessments (N = 130). For both: **(A)** P1: mechanistic understanding of the heart as an effective pump. **(B)** P2: physiological purpose of emergency response to ineffective circulation. The figure presents the distribution of students across conceptual levels, the direction of individual change (improvement, stability, or regression), the magnitude of conceptual change (Δ = POST − PRE), and the results of Wilcoxon signed-rank tests. Conceptual levels range from N0 (non-evaluable response) to N4 (advanced physiological integration). Both assessment dimensions showed statistically significant improvements following the intervention.

In the PRE assessment, students were distributed across conceptual levels as follows: N0 = 6 (4.6%), N1 = 20 (15.4%), N2 = 74 (56.9%), and N3 = 30 (23.1%). No student reached N4. The mean score was 1.98 ± 0.76, with a median of 2.00. In the POST assessment, the distribution was: N0 = 3 (2.3%), N1 = 8 (6.2%), N2 = 62 (47.7%), N3 = 52 (40.0%), and N4 = 5 (3.8%). The mean score increased to 2.37 ± 0.76, while the median remained 2.00. The proportion of students reaching advanced conceptual levels (N3–N4) increased from 23.1% to 43.8% (**Figure 4A**).

#### 3.3.2 P2 – physiological rationale for healthcare intervention following loss of effective blood flow

In the PRE assessment, the distribution was: N0 = 4 (3.1%), N1 = 37 (28.5%), N2 = 84 (64.6%), and N3 = 5 (3.8%). No student reached N4. The mean score was 1.69 ± 0.60, with a median of 2.00. In the POST assessment, the distribution changed to: N0 = 1 (0.8%), N1 = 10 (7.7%), N2 = 49 (37.7%), N3 = 51 (39.2%), and N4 = 19 (14.6%). The mean score increased to 2.59 ± 0.86, and the median increased to 3.00. The proportion of students reaching advanced conceptual levels (N3–N4) increased from 3.8% to 53.8% (**Figure 4B**).

### 3.4 PRE–POST transition patterns

**Figure 5:**
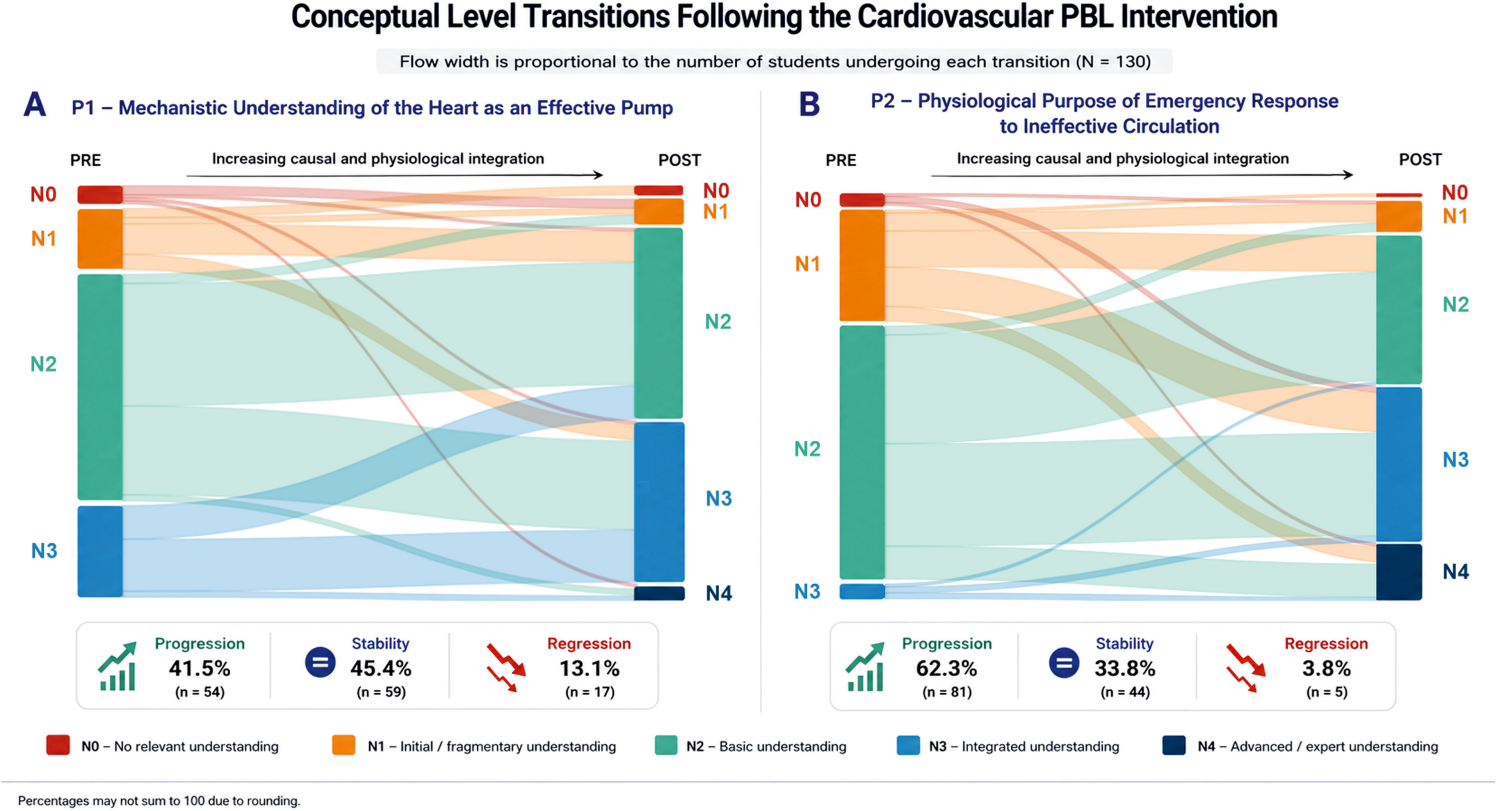
Conceptual level transitions following the cardiovascular PBL intervention. Sankey diagrams illustrating individual transitions between conceptual levels from PRE to POST assessment (N = 130). **(A)** P1: mechanistic understanding of the heart as an effective pump. **(B)** P2: physiological purpose of emergency response to ineffective circulation. Flow width is proportional to the number of students undergoing each transition. Colors represent conceptual levels ranging from N0 (non-relevant understanding) to N4 (advanced/expert understanding). Summary indicators show the proportions of progression, stability, and regression observed in each dimension.

Cross-tabulation analyses were used to identify individual patterns of conceptual change between PRE and POST assessments. Complete transition flows are presented in **Figure 5** and **Supplementary Material 3**.

#### 3.4.1 Transitions in P1

Among students classified as N1 in the PRE assessment (n = 20), 10 progressed to N2 (50.0%), 5 progressed to N3 (25.0%), 2 remained at N1 (10.0%), and 3 regressed to N0 (15.0%). Among students initially classified as N2 (n = 74), 40 remained at N2 (54.1%), 29 progressed to N3 (39.2%), and 2 reached N4 (2.7%), whereas 3 regressed to N1 (4.1%). Of the students initially classified as N3 (n = 30), 17 remained at N3 (56.7%), 2 progressed to N4 (6.7%), and 11 regressed to N2 (36.7%).

#### 3.4.2 Transitions in P2

Among students classified as N1 in the PRE assessment (n = 37), 6 remained at N1 (16.2%), 12 progressed to N2 (32.4%), 13 progressed to N3 (35.1%), and 5 reached N4 (13.5%), while 1 regressed to N0 (2.7%). Students initially classified as N2 (n = 84) showed a predominantly upward transition pattern: 36 remained at N2 (42.9%), 34 progressed to N3 (40.5%), and 11 reached N4 (13.1%), whereas only 3 regressed to N1 (3.6%). Of the five students initially classified as N3, 2 remained at N3 (40.0%), 2 progressed to N4 (40.0%), and 1 regressed to N2 (20.0%).

### 3.5 PRE–POST comparisons using non-parametric analyses

**Figure 6:**
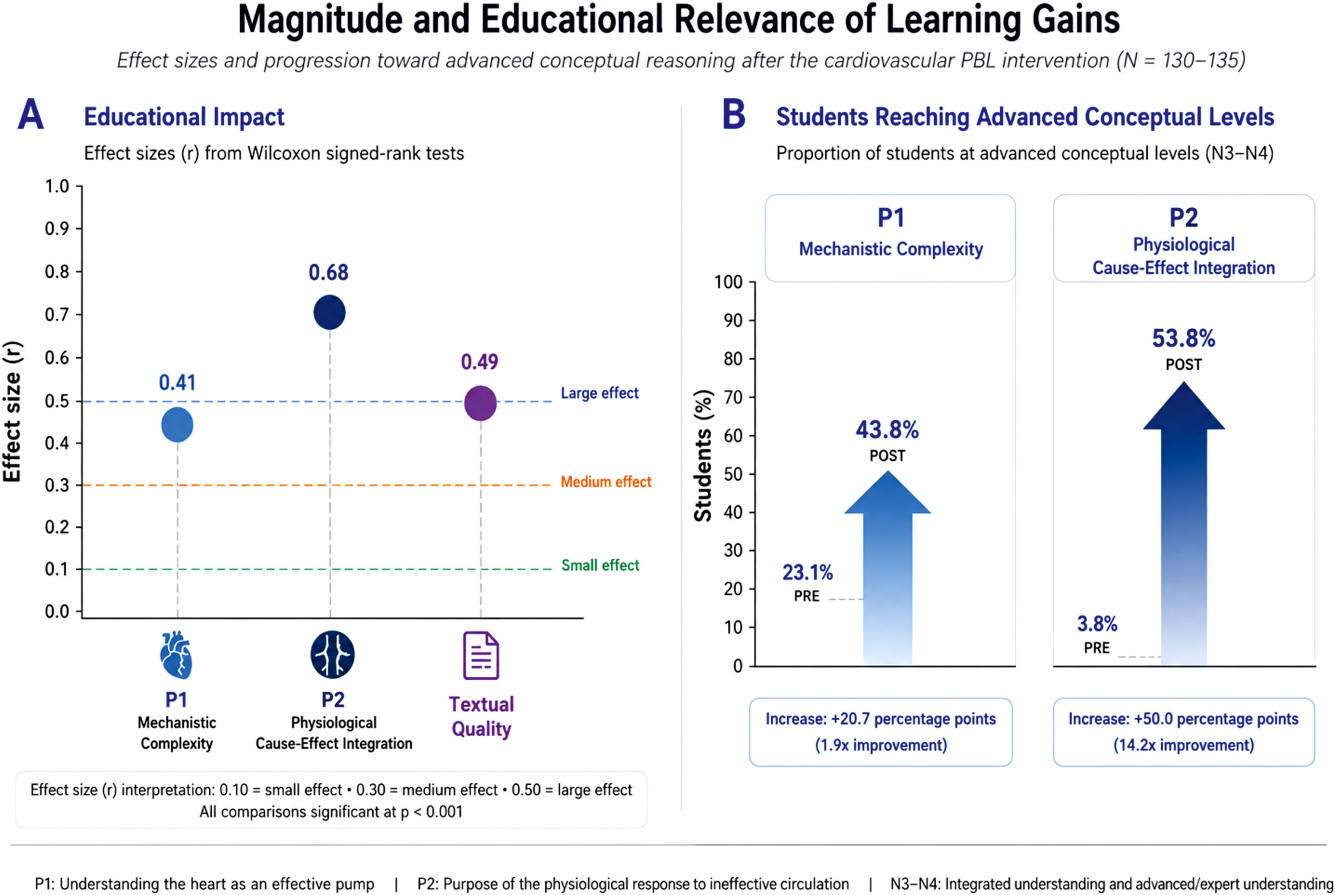
Magnitude and educational relevance of learning gains following the cardiovascular PBL intervention. **(A)** Effect sizes (r) derived from Wilcoxon signed-rank tests comparing PRE and POST performance in mechanistic complexity (P1), physiological cause–effect integration (P2), and overall textual quality. Horizontal reference lines indicate conventional thresholds for small, medium, and large effects. **(B)** Changes in the proportion of students achieving advanced conceptual levels (N3–N4) after the intervention in both assessment dimensions. The figure summarizes the educational significance of the observed learning gains and progression toward higher-order physiological reasoning.

Wilcoxon signed-rank tests revealed significant PRE–POST differences for both P1 and P2 (**Figure 6**). For P1 (N = 130), there were 54 positive ranks, 17 negative ranks, and 59 ties, yielding Z = −4.683, p < 0.001, with an effect size of r = 0.41 (moderate effect). For P2 (N = 130), there were 81 positive ranks, 5 negative ranks, and 44 ties, yielding Z = −7.723, p < 0.001, with an effect size of r = 0.68 (large effect).

Analysis of individual change scores confirmed the direction of improvement. In P1, 33.8% of students increased by one level and 7.7% increased by two or more levels. In P2, 37.7% increased by one level and 24.6% increased by two or more levels.

Spearman’s correlation between changes in P1 and P2 was rs = 0.128 (p > 0.05), indicating that improvements in the two dimensions were independent of one another.

### 3.6 Textual quality of responses

Textual quality (N = 135) improved significantly following the intervention (Wilcoxon signed-rank test: positive ranks = 51, negative ranks = 7, ties = 77; Z = −5.709, p < 0.001, r = 0.49). Overall, 37.8% of students improved their textual quality, 5.2% showed lower scores, and 57.0% remained unchanged.

### 3.7 Student perceptions of the intervention

**Figure 7:**
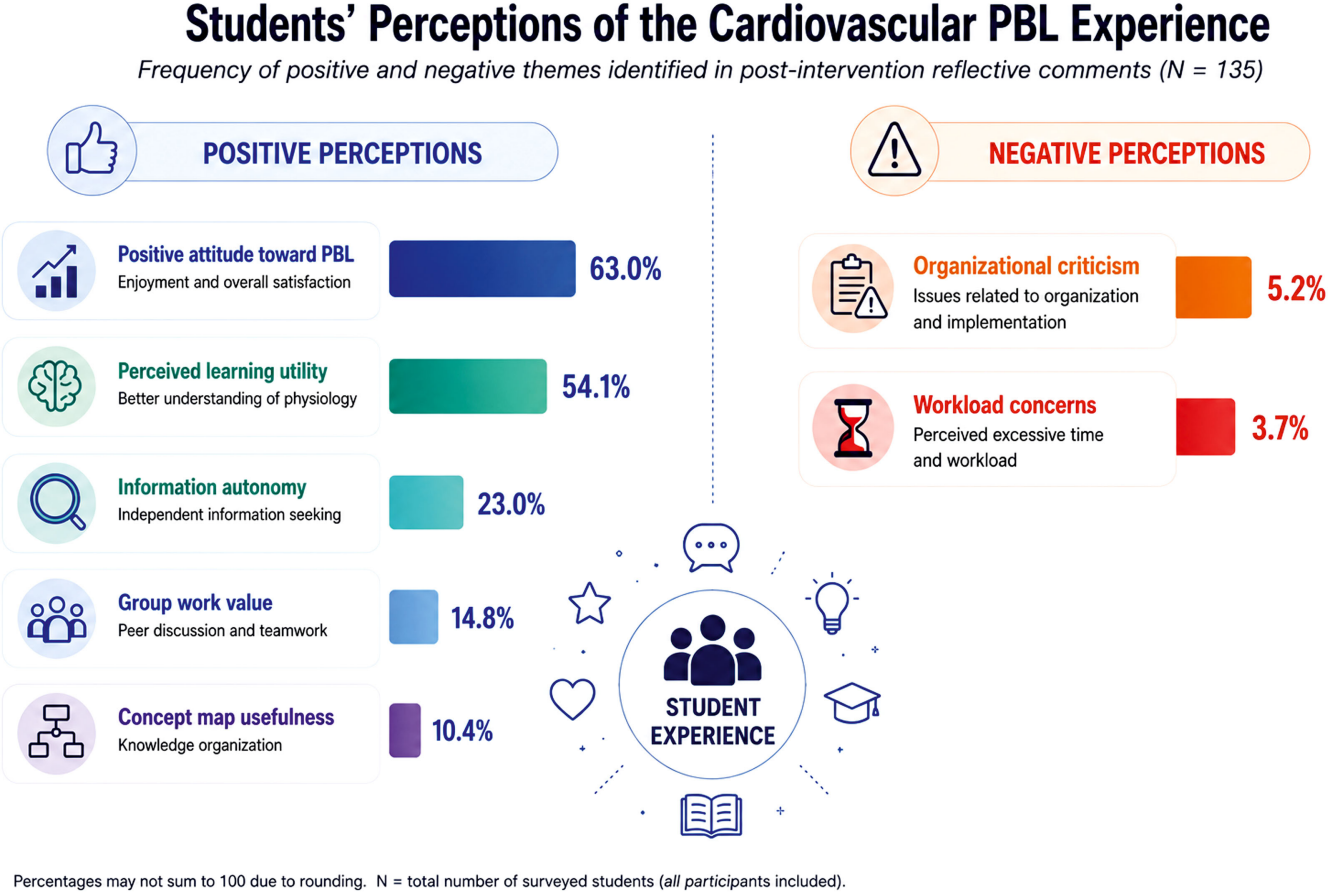
Students’ perceptions of the cardiovascular PBL experience based on post-intervention reflective comments. Frequency of positive and negative themes identified through qualitative content analysis of students’ written reflections following completion of the intervention (N = 135). Positive perceptions included enjoyment and satisfaction with the activity, perceived learning utility, autonomous information seeking, collaborative learning, and usefulness of concept-map construction. Negative perceptions were infrequent and primarily related to organizational aspects and perceived workload. Positive and negative perceptions are displayed around a central representation of the overall student experience. Percentages indicate the proportion of students mentioning each theme.

Student perceptions of the intervention are summarized in **Figure 7**. Most participants expressed a positive overall evaluation of the activity. Eighty-five students (63.0%) provided explicitly positive comments regarding the experience, whereas only two students (1.5%) expressed clearly negative views. The remaining responses did not contain an explicit overall evaluation.

Regarding perceived usefulness for learning, 73 students (54.1%) described the activity as helpful for understanding or consolidating physiological concepts. No explicitly negative comments were recorded in this category. Positive references to group work appeared in 20 responses (14.8%), whereas 3 students (2.2%) reported difficulties related to coordination, unequal participation, or group dynamics. Concept mapping was explicitly identified as a useful learning tool by 14 students (10.4%), with no negative comments recorded for this resource.

Positive references to self-directed learning and information searching were identified in 31 responses (23.0%). Five students (3.7%) described workload or task difficulty as problematic. With regard to the organization and implementation of the activity, seven students (5.2%) expressed criticisms related to structure, available time, or session dynamics. One student (0.7%) made a positive comment regarding organization, and one student (0.7%) expressed a negative comment regarding autonomy. Most remaining responses were classified as neutral or contained no explicit references to the categories analyzed.

## 4 Discussion

The present study examined whether a Problem-Based Learning (PBL) sequence centered on authentic cardiovascular scenarios and implemented during the first year of medical training could improve two conceptually independent dimensions of physiological reasoning: mechanistic conceptual complexity of the cardiac pump model (P1) and physiological cause–effect integration underlying healthcare intervention (P2). Significant improvements were observed in both dimensions, although their magnitudes differed substantially. Physiological cause–effect integration (P2) showed a large effect size (r = 0.68), whereas mechanistic conceptual complexity (P1) demonstrated a moderate effect (r = 0.41). The absence of correlation between improvements in the two dimensions (rs = 0.128, p > 0.05) indicates that they followed partially independent developmental trajectories. This finding supports their assessment as distinct cognitive constructs and represents one of the most relevant outcomes of the study.

### 4.1 Improvements in mechanistic conceptual complexity (P1) and physiological cause–effect integration (P2)

The improvement observed in P1, although statistically robust, was moderate in magnitude. This result is consistent with the initial distribution of the sample: more than half of the students were already classified at N2 before the intervention, and nearly one quarter had reached N3. Consequently, the potential for upward progression was limited for a substantial proportion of participants. Nevertheless, the proportion of students reaching advanced conceptual levels (N3–N4) nearly doubled, and 41.5% of students demonstrated some degree of progression. P1 addressed the physiological functioning of the heart as an effective pump, a topic that students are likely to perceive as relatively familiar from prior educational experiences. The moderate effect may therefore reflect a partial ceiling effect rather than a limitation of the intervention itself. This interpretation is consistent with findings from active-learning interventions in pathophysiology, where the strongest gains are typically observed in tasks requiring application and clinical integration according to Bloom’s taxonomy (Cen et al., 2025). Such characteristics closely resemble those assessed in P2, which has been identified as particularly sensitive to active-learning approaches in cardiovascular physiology (Wafi, 2025).

The most striking result of the study was the large effect observed in P2. At baseline, only a small number of students demonstrated explicit causal integration (N3), and none reached N4. Following the intervention, more than half of the cohort achieved N3 or N4 levels. The transition from an almost complete absence of integrated physiological reasoning to a majority of students demonstrating such reasoning represents a substantial educational shift following a relatively brief intervention.

Unlike P1, P2 addressed content that was genuinely novel for most participants. The pathophysiology of cardiac arrest and the mechanistic rationale underlying cardiopulmonary resuscitation and related interventions are not generally part of students’ intuitive prior knowledge. The cardiovascular collapse cases involving Christian Eriksen and Damar Hamlin, including documented CPR and defibrillation procedures, served as powerful triggers for inquiry directly aligned with the concepts assessed by P2. Confronting students with authentic loss-of-perfusion scenarios required them to actively construct physiological causal chains linking pump failure, impaired tissue perfusion, systemic consequences, and the rationale for intervention—precisely the form of reasoning captured by the N3–N4 levels of the coding framework (Lira et al., 2024). This finding is consistent with previous evidence from first-year physiology education. Gaddas et al. (2026) reported retention differences of up to 50 percentage points between inquiry-based learning and team-based learning in preclinical physiology, a magnitude comparable to the transformation in conceptual-level distribution observed in P2. Such findings suggest that authentic clinical problems may facilitate cognitive restructuring when students encounter genuinely unfamiliar physiological content (Langer et al., 2021; Vieira, 2025).

The absence of correlation between changes in P1 and P2 further suggests that different dimensions of physiological reasoning may develop independently. This interpretation aligns with the distinction proposed by Lira et al. (2024) between proximate-How reasoning (mechanistic description of physiological processes) and proximate-Why reasoning (functional cause–effect integration), which have been shown to respond differently to instructional prompts and scaffolding strategies. The present results extend these observations by demonstrating that gains in one dimension do not necessarily predict gains in the other.

This finding also constitutes preliminary evidence for the construct validity of the N0–N4 framework: the absence of correlation between improvements in two theoretically distinct dimensions provides empirical support for the framework’s capacity to discriminate between independent cognitive constructs. Accordingly, future educational interventions may benefit from targeting these dimensions separately depending on learners’ initial profiles and instructional objectives.

### 4.2 Textual quality

Textual quality also improved significantly following the intervention. More than one third of students (37.8%) improved the overall quality of their written responses, whereas only 5.2% showed lower scores. This finding is consistent with evidence indicating that PBL simultaneously promotes critical thinking and communication skills through collaborative problem solving (Abugri et al., 2026). Similarly, Saudagar and Madadin (2025) reported that more than 92% of students perceived improvements in communication and presentation skills after participating in PBL activities. However, these gains should be interpreted primarily as reflecting increased familiarity with physiological terminology and explanatory discourse acquired during the self-directed inquiry phase rather than as direct evidence of deeper conceptual understanding. Importantly, the N0–N4 coding framework was specifically designed to distinguish conceptual complexity from expressive fluency, supporting the interpretation that improvements in P1 and P2 cannot be explained solely by enhanced writing performance.

### 4.3 Comparison with previous literature

The effect sizes observed in the present study are consistent with, and in the case of P2 exceed, those reported in comparable educational interventions. Sharma et al. (2023), in a meta-analysis of 16 studies on PBL in nursing education, reported a standardized mean difference (SMD) of 0.44 for overall critical thinking and 0.72 for analytical reasoning. Although SMD and r represent different statistical metrics, both provide useful benchmarks against which the present effects for P1 (r = 0.41) and P2 (r = 0.68) can be interpreted.

Mensour et al. (2025) found that PBL and traditional instruction produced similar outcomes when assessed through conventional multiple-choice examinations. The stronger effects observed here, particularly for P2, may therefore reflect the greater sensitivity of the ordinal coding framework to detect dimensions of causal integration that conventional assessment instruments often fail to capture. This interpretation is further supported by the convergence between our objective coding of physiological reasoning and previous reports based on students’ perceived confidence in clinical reasoning (Kirchberg et al., 2026).

Although the absence of an independent control group prevents causal attribution of the observed changes exclusively to the intervention, the magnitude of the shift in P2—from a distribution concentrated in N1–N2 levels to one dominated by N3–N4 reasoning—appears unlikely to be explained solely by the passage of time or concurrent lecture-based instruction, given both the specificity of the content and the short timeframe involved.

### 4.4 Implications for curriculum design

This study forms part of a broader research program within our Department of Human Physiology focused on innovative active-learning methodologies in medical physiology education, including previous experiences with educational escape rooms (Carrasco-Gomez et al., 2023) and service-learning approaches in non-formal educational environments (García-Durán et al., 2023). The present PBL intervention extends this portfolio by incorporating authentic cardiovascular clinical scenarios together with a structured framework for assessing physiological reasoning.

The findings support the integration of PBL modules into preclinical basic science curricula, particularly when organized around authentic clinical problems that require students to construct physiological causal chains actively (Langer et al., 2021; Lira et al., 2024). The effectiveness of the intervention during the first year of medical training—before formal lecture-based instruction in cardiovascular physiology—may be especially relevant. It suggests that early exposure to authentic clinical cases is not only feasible but can promote meaningful development of causal physiological reasoning even in learners without extensive prior biomedical knowledge.

The intervention was also operationally simple, requiring only two three-hour face-to-face sessions and small groups of five students, without additional infrastructure. This feasibility is consistent with findings reported by Uqaili et al. (2026), who demonstrated that case-based learning in preclinical physiology can generate meaningful educational benefits even in resource-limited settings when facilitation is appropriately standardized. Furthermore, the face-to-face format preserved the social dimension of PBL, including collaborative discussion and knowledge co-construction, which remains one of its defining educational features (Rathleff et al., 2025).

The present findings should be interpreted in light of their institutional and disciplinary context. The cognitive processes targeted — constructing causal chains linking physiological mechanisms to their functional consequences — are not unique to cardiovascular physiology and have been shown to respond to scaffold-based instruction across multiple physiology domains (Lira et al., 2024). However, the trigger scenarios employed here may carry exceptional clinical salience: the documented real-time cardiovascular collapses of professional athletes, involving CPR and defibrillation, provided an unusually immediate and emotionally engaging clinical context. Whether comparable gains in causal physiological reasoning would be observed in domains with less intuitively relatable clinical triggers — such as endocrine or renal physiology — or in settings with different class sizes, facilitation resources, or prior active-learning exposure, remains an open empirical question that future comparative studies should address.

### 4.5 Limitations

A primary limitation of the present study is the absence of an independent control group. All participants were exposed to the PBL intervention, and the concurrent delivery of conventional lecture-based teaching on cardiovascular physiology throughout the same period introduces a confounding variable that cannot be fully disentangled from the effects of the intervention itself. Accordingly, the improvements observed in both P1 and P2 should be interpreted as outcomes of the overall contextualized educational sequence rather than as the isolated effect of the PBL sessions. Future studies incorporating a parallel control condition—whether a matched cohort receiving conventional instruction only or a waitlist design—would allow more precise estimation of the specific contribution of PBL to the development of causal physiological reasoning.

The inter-rater reliability obtained (κ = 0.39–0.52) reflects fair to moderate agreement, consistent with published benchmarks for ordinal instruments of comparable complexity in clinical reasoning assessment (Valentine et al., 2022), and the involvement of a third adjudicating evaluator provided a procedural safeguard against rater inconsistency. Findings should therefore be interpreted primarily at the group level, as indicators of collective conceptual trajectories rather than as precise individual classifications.

A further limitation of the study concerns the potential influence of social desirability bias on students’ written reflections. Because participants were aware that instructors would have access to their comments, positive evaluations may have been overrepresented. For this reason, qualitative perception data were interpreted conservatively, with particular attention paid to critical and negative comments. In contrast, conceptual progression outcomes were derived from independent blind coding procedures and therefore constitute a more objective source of evidence.

The study also lacked longitudinal follow-up. Csaba et al. (2025) demonstrated that physiology knowledge may decline by approximately 25% within 16 weeks under conventional instructional conditions. Future studies should therefore incorporate retention assessments to determine the long-term stability of the gains observed.

An additional limitation concerns variability in cooperative group functioning. Li et al. (2022) reported that group dynamics predict experiential outcomes more consistently than academic performance, making it difficult to determine the extent to which observed gains were influenced by the specific quality of each group’s interaction. Future research should incorporate procedures that provide greater guarantees of anonymity when collecting student perceptions, such as external survey platforms, or include indirect measures of engagement and motivation that do not rely exclusively on self-report.

Finally, POST responses were collected immediately after the final discussion session, under greater time pressure than the PRE assessment, which was completed in a large-group setting with sufficient time available. This asymmetry may have led some students to produce shorter responses that, although conceptually correct, omitted mechanistic details required to achieve N3 classification. Such circumstances may partially explain the regressions observed in a subset of participants, particularly within P1.

## 5 Conclusions

A brief Problem-Based Learning (PBL) intervention centered on authentic cardiovascular scenarios produced significant improvements in physiological reasoning among first-year medical students before the formal introduction of cardiovascular physiology lectures. The most notable finding was the large effect observed for physiological cause–effect integration (P2, r = 0.68), a dimension of reasoning that was largely absent at baseline and showed substantial development following the intervention. The fact that two small-group sessions, implemented without additional infrastructure, generated improvements that were independent of gains in mechanistic conceptual complexity (P1) suggests that early exposure to authentic clinical cases can promote the development of distinct dimensions of physiological reasoning during the preclinical stage of medical education.

The N0–N4 coding framework developed in this study was designed to discriminate between mechanistic conceptual complexity and physiological cause–effect integration in open-ended written responses. The absence of correlation between gains in these two dimensions provides preliminary evidence that the framework captures theoretically distinct cognitive constructs — a finding that supports its potential as an instrument sensitive to the multidimensional nature of physiological reasoning development. Its structure may provide a transferable approach for evaluating causal physiological reasoning across other physiology domains, such as respiratory, renal, or endocrine physiology, and, with appropriate adaptation, in broader health sciences education contexts where conceptual progression is assessed within active-learning environments. External validation through independent application by raters outside the original research group, and replication across physiology domains beyond cardiovascular physiology, remain important next steps before the framework can be regarded as a fully established assessment instrument.

## Data Availability

The raw data supporting the conclusions of this article will be made available by the authors, without undue reservation.

## Supplementary material

Supplementary Figure 1 Emergent classroom whiteboard generated during the collaborative brainstorming phase. Representative example of the collective whiteboard developed by students during the initial exploration of the clinical scenario. The board contains hypotheses, questions, causal relationships, and learning objectives identified by the groups before independent information gathering. It illustrates the emergence of shared conceptual structures and the formulation of research questions that guided subsequent learning activities.

Supplementary Figure 2 Representative student-generated concept map produced after autonomous information seeking. Example of an individual concept map developed during the second PBL session. The map integrates physiological concepts, risk factors, diagnostic approaches, prevention strategies, treatment options, and recovery considerations associated with cardiovascular events in athletes. The figure illustrates how students organized and interconnected knowledge acquired throughout the learning process.

Supplementary Figure 3 Representative conceptual level transition matrices from PRE to POST assessment. Transition matrices showing the number and percentage of students moving between conceptual levels in P1 (understanding of the heart as an effective pump) and P2 (physiological purpose of emergency response to ineffective circulation). Rows represent PRE levels and columns represent POST levels. The matrices provide a detailed view of individual conceptual trajectories underlying the overall learning gains observed after the intervention.
