# Supplementary figures and images for: Problem-based learning promotes causal physiological reasoning in first-year medical students before formal cardiovascular instruction
Source: Front Physiol. 2026 Jul 17;17:1897473. doi: 10.3389/fphys.2026.1897473 (PMC13423646; doi:10.3389/fphys.2026.1897473)

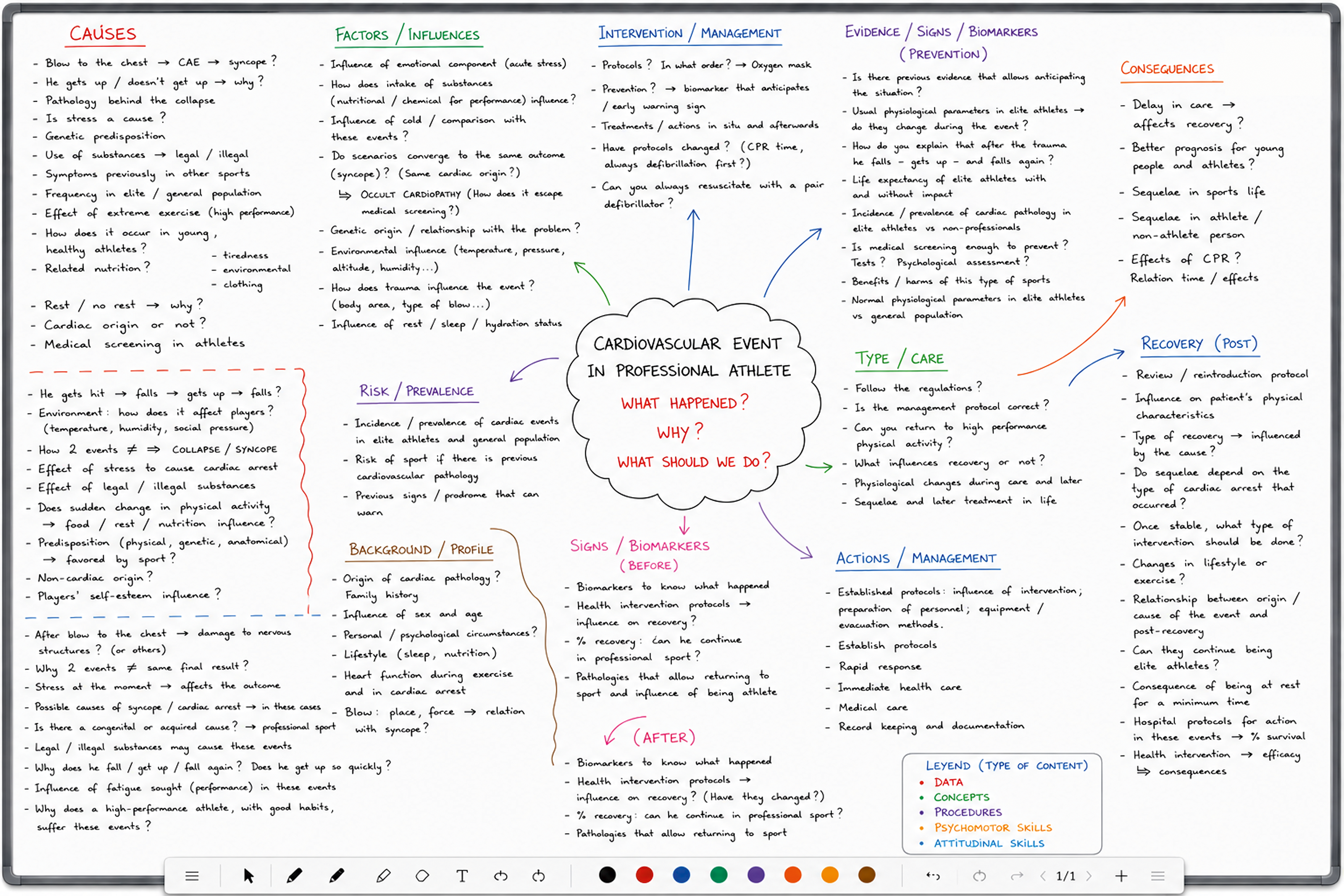

Supplement: Supplementary Figure 1 — Emergent classroom whiteboard generated during the collaborative brainstorming phase. Representative example of the collective whiteboard developed by students during the initial exploration of the clinical scenario. The board contains hypotheses, questions, causal relationships, and learning objectives identified by the groups before independent information gathering. It illustrates the emergence of shared conceptual structures and the formulation of research questions that guided subsequent learning activities. [file Image1.jpeg]

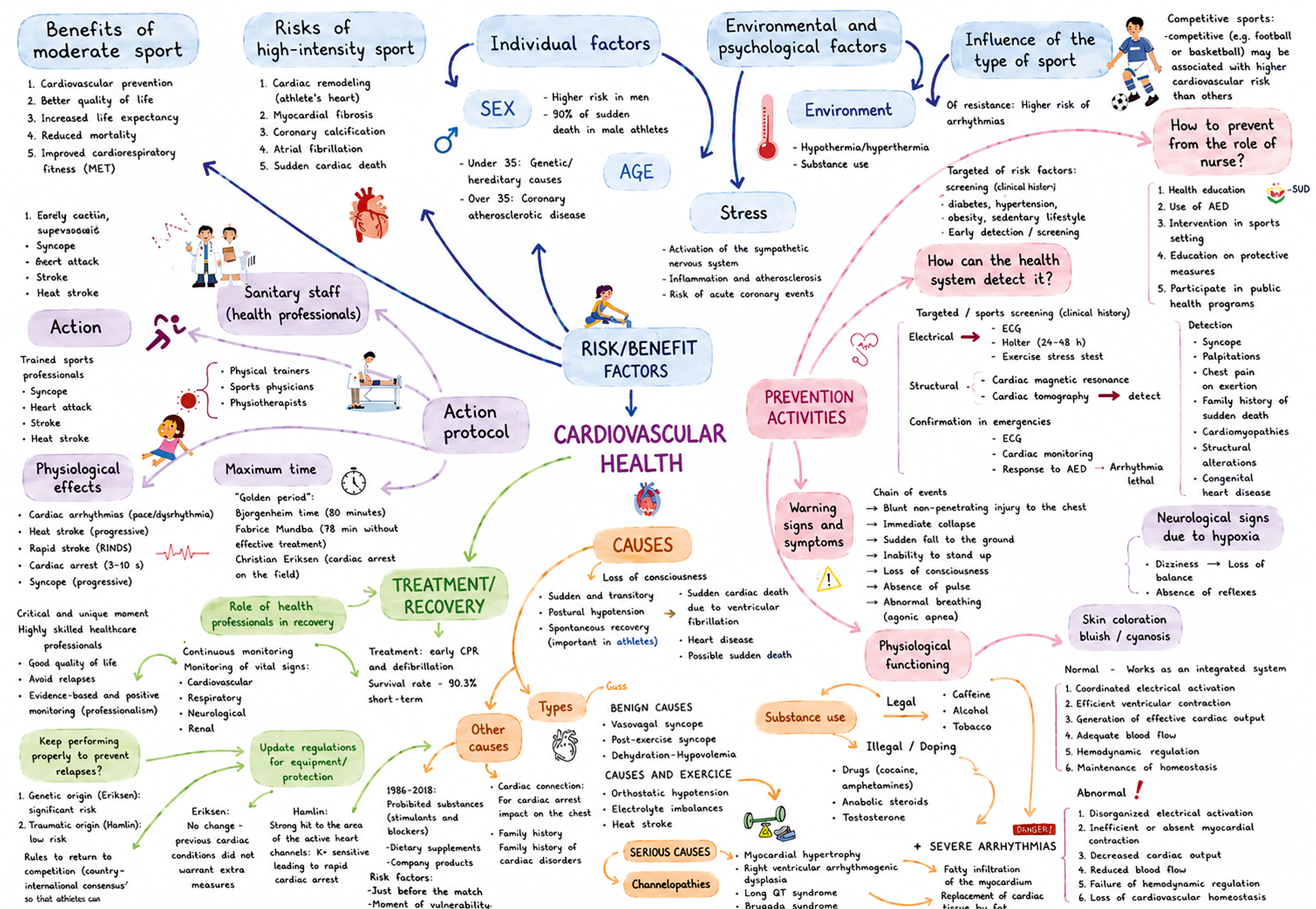

Supplement: Supplementary Figure 2 — Representative student-generated concept map produced after autonomous information seeking. Example of an individual concept map developed during the second PBL session. The map integrates physiological concepts, risk factors, diagnostic approaches, prevention strategies, treatment options, and recovery considerations associated with cardiovascular events in athletes. The figure illustrates how students organized and interconnected knowledge acquired throughout the learning process. [file Image2.jpeg]

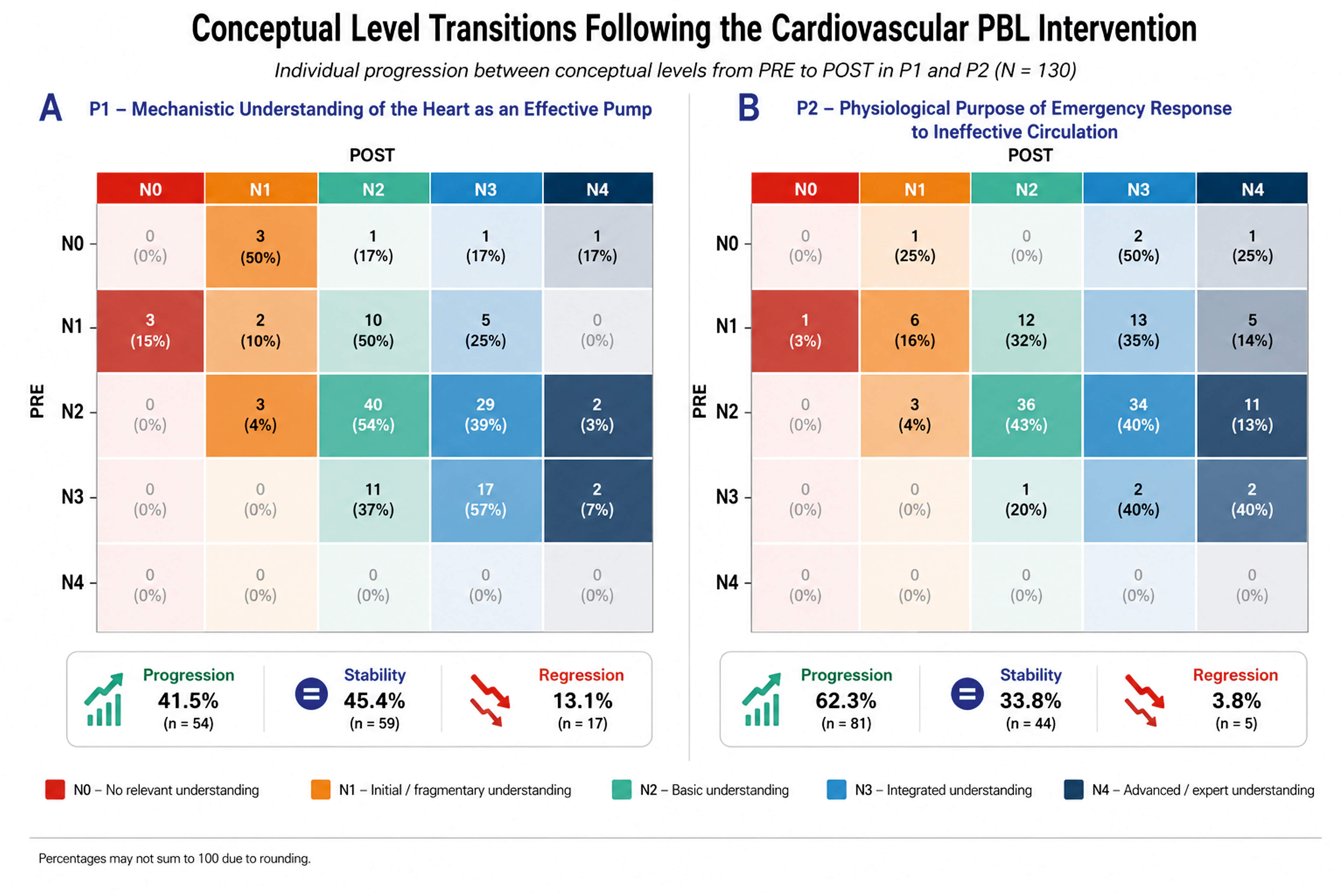

Supplement: Supplementary Figure 3 — Representative conceptual level transition matrices from PRE to POST assessment. Transition matrices showing the number and percentage of students moving between conceptual levels in P1 (understanding of the heart as an effective pump) and P2 (physiological purpose of emergency response to ineffective circulation). Rows represent PRE levels and columns represent POST levels. The matrices provide a detailed view of individual conceptual trajectories underlying the overall learning gains observed after the intervention. [file Image3.jpeg]
